# Soft lithography-based biomolecule patterning techniques and their applications in subcellular protein interaction analysis

**DOI:** 10.1016/j.mtbio.2025.101672

**Published:** 2025-03-17

**Authors:** Tina Karimian, Peter Lanzerstorfer, Julian Weghuber

**Affiliations:** a University of Applied Sciences Upper Austria, Center of Excellence Food Technology and Nutrition, Stelzhamerstrasse 23, 4600, Wels, Austria; b FFoQSI GmbH-Austrian Competence Centre for Feed and Food Quality, Safety and Innovation, Stelzhamerstrasse 23, 3430, Tulln, Austria

**Keywords:** Soft lithography contact printing, Protein patterning, Subcellular micropatterning, Protein-protein-interactions

## Abstract

Soft lithography-based contact printing techniques have evolved into versatile methods for creating micro- and nanoscale features of biomolecules on solid substrates. In this review we present the advances in soft lithography for biomolecule deposition and its applications in subcellular protein-protein interaction (PPI) analysis. We discuss various soft lithography techniques, including micro-contact printing (μCP), nano-contact printing (nCP), capillary nanostamping, and polymer-pen-lithography (PPL) and focus on their application in biomolecule patterning on diverse substrates. We then address related challenges and advancements, including substrate selection, surface activation methods, and stamp development. The specific advantages, limitations, and potential solutions for printing various inks and biomolecules are highlighted. Furthermore, recent advances in soft lithography-based biomolecule patterning for subcellular protein interaction analysis are emphasized, demonstrating the importance of these techniques for incorporating complex cellular events into PPI readout modalities and established protein deposition strategies. Finally, an overview of future technologies and enhanced applications is provided.

## Graphical abstract

**Figure:**
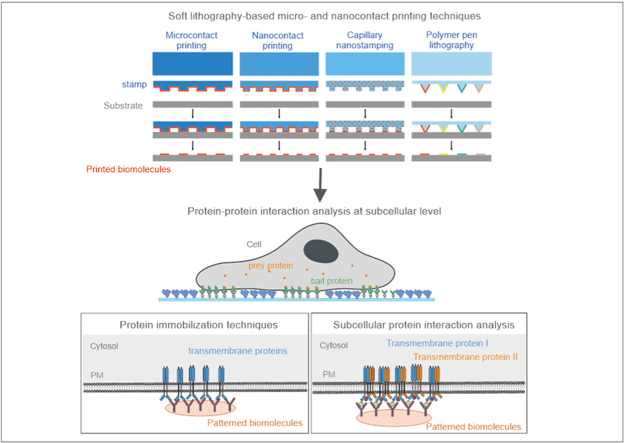

**Statement of Significance**

This review highlights the potential of soft lithography-based printing for biomolecule patterning, focusing on micro-contact printing (μCP), nano-contact printing (nCP), capillary nanostamping, and polymer-pen-lithography (PPL) and their application in investigating protein-protein interactions (PPIs) at the subcellular level. These methods allow for precise deposition of biomolecules at the micro- and nanoscale, advancing the investigation of intricate cellular interactions. By exploring the latest improvements and addressing current challenges of each printing method, this review offers valuable insights for utilization of soft lithography-based printing methods for subcellular PPI studies in cellular environment. Our work attempts to expand our understanding of cellular mechanisms and advancing the development of biomedical diagnostics and therapies.

## 1 Introduction

For centuries, biomolecule patterning techniques have been used for various purposes and only in recent decades, these surface modification approached have been optimized for the production of micro- and nanometer sized structures on solid surfaces [[1], [2], [3]]. Various techniques for creating micro- and nanometer sized arrangements exist, including photolithography [4], ink-jet printing [5], laser micro-ablation [6], dip pen nanolithography [7] and soft lithography-based contact printing [8,9]. Among these printing methods, soft lithography is recognized as a versatile and innovative technique. In this technique, elastomeric stamps are used due to their unique advantages in scalability, cost-effectiveness, and compatibility with various substrate materials. Here we explain the principles and biological applications of different soft lithography-based contact printing methods.

Microcontact printing (μCP) was initially developed by Whitesides et al. in the 1990s and is known for creating molecular patterns in micrometer-range resolution from elastomeric stamp to the substrate [[10], [11], [12]]. Similar to μCP, in nano-contact printing (nCP), proteins are loaded on the elastomeric stamp and are printed on the surface with nanometer resolution [13]. In capillary nanostamping, the loaded ink or biomolecules on the stamp are transferred to the substrate using the capillary movement creating structures with sub-100 nm resolution [14]. In polymer-pen-lithography (PPL), an elastomeric stamp is secured on a high-precision device to allow nanoscale printing resolution, multiple stamp cycles without reinking and biomolecule multiplexing [15].

These soft lithography-based techniques facilitate investigations of cell adhesion, migration, differentiation, and signalling, providing valuable insights in various research fields including cell biology, tissue engineering, and biosensing [8,[16], [17], [18]]. These methods have been used to fabricate biosensors and lab-on-chip technologies, offering precise control of the spatial distribution of biomolecules [[19], [20], [21], [22]]. Created patterned surfaces enable protein immobilization and reorganization of membrane proteins in living cells, assisting in investigating physiological changes and gaining insight into PPI analysis [23,24].

Many reviews exist exploring the application of soft lithography-based printing techniques in cell morphology, differentiation and migration studies [8,16,23,25]. However, their related applications for subcellular protein interaction analysis have not been specifically covered. In this comprehensive review we aim at highlighting the study of soft lithography-based micro and nano patterning techniques adapted to subcellular PPI analysis and at supporting future research in this field.

### 1.1 Contact printing for biomolecule patterning – a brief history

Initial attempts at biomolecule patterning, such as hand-pipetting [26] and brush application [27], were introduced to manually create biomolecule patterns on surfaces. These early techniques were time-consuming and lacked the precision required for detailed applications. The introduction of microcontact printing in the early 90s allowed producing controlled repetitive patterns on solid surfaces using elastomeric stamps [12]. This invention enabled the creation of well-defined patterns with micrometer-scale resolution and was initially used to pattern self-assembled ink monolayers, later extended to printing biomolecules [10].

### 1.2 Developments in biomolecule patterning

Over time, contact printing improved by optimizing ink formulations and substrate materials to increase the precision of ink transfer and to develop control factors such as stamp deformation, ink diffusion, and substrate activation [28]. Consequently, many researchers enhanced contact printing techniques by achieving nanometer-scale structure resolution, generating multicomponent patterning, and complex biomolecular architectures on surfaces [1,29,30]. As contact printing matured, researchers focused on utilizing these high-resolution printing techniques to produce patterned surfaces suitable for biological applications to study cell behaviour, biosensors, and biochip development. In many studies micro- and nano-contact printing were combined with other techniques, such as dip-pen lithography and layer-by-layer assembly, to produce higher resolution and more complex pattern architecture [[31], [32], [33], [34]]. These techniques create intricate biomolecular arrays with diverse functionalities and applications in various research fields [35]. Nowadays, the field continues to evolve, focusing on achieving more complex structures and enhancing control and specificity over pattern production.

### 1.3 Stamp materials used in biomolecule patterning and their properties

**Fig. 1:**
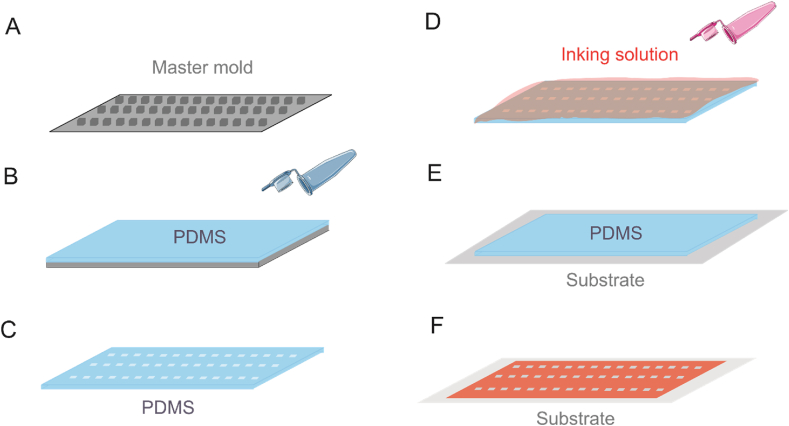
Schematic of producing stamp for soft lithography-based contact printing. A) A master mold is created. B, C) Liquid PDMS is poured onto the master mold, cured and then peeled off, in a negative pattern of the master. D, E) Ink or biomolecules are applied to the patterned PDMS stamp, covering its top layer. The loaded stamp is washed and placed on the substrate. F) After the PDMS is removed, the patterned biomolecules are transferred to the substrate.

In soft lithography-based contact printing techniques mostly an elastomeric stamp is used for creating biomolecule patterns on solid surfaces. Polydimethylsiloxane (PDMS) is the preferred material for the fabrication of micro and nano-scale structures on the stamp [10,36]. The production of these stamps involves using a master mold with the desired micro- or nanoscale pattern (Fig. 1). Liquid PDMS is poured and cured onto this master, then peeled off as a negative replica of the pattern. The PDMS stamp is typically coated with ink, biomolecules or functionalized with specific chemical groups to facilitate binding [[37], [38], [39]]. When it comes into contact with the surface, patten of biomolecules are transferred to the substrate. The stamp is then carefully lifted off, leaving behind the patterned biomolecules.

PDMS stamps are cost-effective, and the procedure is straightforward and adaptable. They can be operated under mild processing conditions and are suitable for the direct patterning of proteins and other biomolecules, with reduced denaturation or loss of bioactivity [8,38]. The minimal mechanical stress during patterning ensures a non-destructive process. Additionally, the adaptability of stamp design allows great flexibility in tailoring patterns according to specific experimental requirements and resolution (down to sub-100 nm). Some well-known derivatives are magnetic PDMS [40], pyramidal PDMS [41], AuNPs–PDMS [42], and X-PDMS, which consists of a two-layer of Vinyl Q-siloxane and Vinyl linear siloxane, covered with a 2 mm PDMS layer on top [43].

However, PDMS is hydrophobic, meaning environmental molecules and dust may be absorbed during handling, potentially contaminating the surface. Contaminants can interfere with structures, making the method unsuitable for patterning molecules requiring high purity. Additionally, stamps tend to wear and deform over time, losing accuracy and resolution. Consequently, stamps may need regular replacement or refurbishment [43]. PDMS may also be unsuitable for specific classes of proteins or molecules that interact unfavourably with the PDMS surface, limiting its use in some contexts [44]. Moreover, achieving precise multi-component patterning with PDMS-based contact printing of different biomolecules with varying chemical and physical properties can also be challenging [45]. The PDMS surface exhibits limited control over specific surface chemistries, limiting its application for biomolecule immobilization on such surfaces [10,19,36].

Due to its unique properties, PDMS is mostly chosen for creating biomolecule patterns for subcellular interaction studies [8,19]. However, alternative stamp materials have been used for printing inks or biomolecules on the surface. For instance, polyolefin stamp allow printing with high structural resolution down to 100 nm. However, its rigid structure makes it suitable only for printing on flat surfaces [1]. Hydrogel stamps provide hydrophilic surfaces to minimize unspecific interactions. They are reusable and are utilized to print ∼20 μm size structures. On the other hand, these stamps require high humidity during curing and printing to prevent deformation of the structures [46,47]. Perfluoropolyether (PFPE) based-elastomeric stamps have been reported to produce homogeneous micrometer-scale pattern structures on large substrates. Their low surface energy makes them suitable for printing variety types of biomolecules [45,48,49]. Spongy mesoporous silica stamps are used in capillary nanostamping to print ink and proteins on the substrate. The unique porous texture allows for repeated printing without re-inking and achieving sub-100nm resolution [14,50]. However, for optimum structure transfer, specific substrate functionalization is necessary [51].

Each stamp material provides unique advantages and limitations (Table 1). By understanding the factors and specific requirements of the experiment, the best suited stamp material can be chosen.

**Table 1:** Comparison of the most used stamp materials for soft lithography-based contact printing.

Stamp material	Resolution	Cost	Scalability	Advantages	limitations
PDMS	Sub-100 nm	Low	High	Cost-effective, easy handling, adaptable, flexible texture, mild processing, minimized protein denaturation	Hydrophobic, can cause contamination, wears over time, not suitable for printing <1 μm structures
Polyolefin	∼100 nm	Moderate	only flat surfaces	High stability, high resolution, no contamination, compatible with variety of substrates	Complex and expensive fabrication, limited ink retention, require proper substrate functionalization
Hydrogel	∼20 μm	Moderate	moderate	Hydrophilic, reusable	Humidity-sensitive, limited resolution
PFPE based-elastomeric stamps	Larger than ∼1 μm	High	High	Low surface energy, suitable for large areas	Expensive,
Spongy porous silica	Sub-100 nm	High	Moderate	Reusable, high resolution, multiple printing without reinking, suitable for large areas	Complicated fabrication process, limited application for biomolecules, requires specific substrate functionalization

### 1.4 Substrates and chemical functionalization

Substrate selection and chemical functionalization play pivotal roles in achieving precise and controlled patterning of micro- and nanometer features on solid surfaces. Glass is widely used due to its stable and smooth surface, providing optimal contact during printing [11,36]. Its availability and compatibility with various printing methods make it a suitable substrate choice [28,52]. Furthermore, silicon substrates are also valuable due to their stability and compatibility with many surface functionalization materials [15,27,29,36,53]. Moreover, polymeric foils have been reported, particularly beneficial because of their flexibility, low costs and ease of shaping [36,54]. These flexible substrates have been used to study and control the traction force between cells and substrate. The deformation of substrate is used as a parameter for measuring cellular traction forces [25].

Unmodified surfaces are rarely used directly for biomolecule patterning, as preceding surface functionalization and activation enhances control over biomolecule immobilization and orientation. Numerous functionalization strategies and chemicals, including epoxy silane, provide highly reactive surfaces for covalent protein binding [45,55,56,56]. Epoxy groups react with hydroxyl groups on the substrate surface, forming covalent bonds that strengthen protein adhesion [13]. However, producing these surfaces can be time-consuming, often leading to non-homogeneous coating. Commercially available streptavidin-coated slides have also been used in combination with μCP [57,58]. These surfaces can be passivated using direct printing strategy to avoid unspecific binding, followed by subsequent functionalization and protein immobilization steps of biotinylated biomolecules.

Various chemical modification techniques are required to improve ink or protein immobilization, adjusting surface energy or hydrophobicity to facilitate precise protein attachment [[59], [60], [61],^61^]. 3-aminopropyltrimethoxysilane (APTMS) or related compounds have been employed to introduce amino groups to glass substrates [28,52,62]. This technique is enhanced when the substrates are pre-activated by plasma treatment [54,63]. Reactive gases such as oxygen and nitrogen create active sites on the surface of the substrate, thereby improving adhesion. Furthermore, plasma activation increases the surface energy for better ink adhesion to polymer and glass substrates [33,52,55,64]. N-Hydroxysuccinimide (NHS) ester chemistry is another alternative for specific protein binding to amine-activated surfaces. Introduced NHS ester groups interact with primary amines on the solid substrate, allowing molecules to attach to amino groups [32]. Polymer-metal-based coatings, developed over time, cover substrates with a metal layer (e.g., gold or titanium) to enhance protein adhesion [43,[65], [66], [67]]. Additionally, a polymer metal-ion-based coating reagent can be used for producing highly activated surfaces [43,65]. This reagent is non-toxic, reusable, and produces highly activated surfaces for protein binding. Moreover, self-assembled monolayers (SAMs) deposit a monolayer of organic molecules, such as alkyltrichlorosilane, on the substrate for controlled and tailored surface chemistry, enabling effective ink transfer [10].

Bovine serum albumin (BSA) is mostly used as the passivating protein due to its stability and for providing an anchor point for cell adhesion [11,52,68]. Alternatively, poly(L-lysine)-grafted-poly(ethylene glycol) (PLL-g-PEG) provides high repellence, reducing the level of unspecific bindings [40,69]. However, Pll-g-PEG functionalized surfaces prevent cell adhesion and might be unsuitable as a passivating structure in cell-based studies. Such unfavorable conditions can be improved by using cell adhesion promoting biomolecules such as fibronectin [33,40] or unspecific proteins [70,71].

## 2 Soft lithography-based contact printing techniques

Soft lithography-based patterning techniques, such as μCP, nCP, capillary nanostamping, and PPL are being used for creating micro- and nanostructures on solid substrates. Each technique has its unique properties that make them advantageous or less favored to the other. μCP and nCP are easy to handle and do not need expensive equipment for printing micro- and nanostructures [1,46]. On the other hand, capillary nanostamping and PPL require specific equipment to provide more flexibility and control in printing [16,51]. This section summarizes the fundamental principles, advancements and limitations of each method and accent the recent innovations in each technique.

### 2.1 Microcontact printing (μCP)

**Fig. 2:**
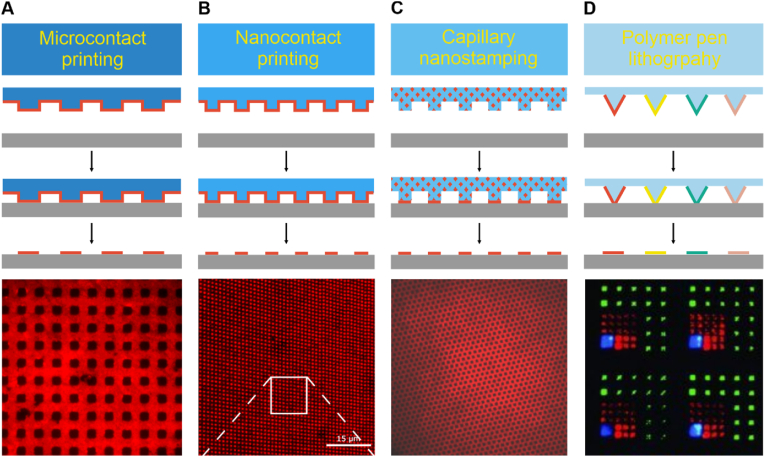
Schematic representation of soft lithography-based contact printing methods. **A)** In μCP, a patterned elastomeric stamp is used to transfer proteins onto the surface with micrometer-scale precision [65], **B)** nCP is similar to μCP, with the difference of using nanoscale stamps for higher deposition resolution down to nanometer scales [74], **C)** in capillary nanostamping, proteins are deposited through capillary forces, allowing precise control over location and density [14]. **D)** polymer pen lithography printing uses an array of polymer pens to deliver biomolecules to the surface, offering scalable, high-precision patterning from the micro-to nanoscale [75]. Each method offers unique features in resolution and protein deposition for producing patterned surfaces. Part A is adapted from Ref. [65] with permission from MDPI, Biosensors; Part B is adapted from Ref. [74] with permission from American Chemical Society, Langmuir; Part C and D are adapted from Refs. [14,75] with permission from John Wiley & Sons, Small.

μCP is significantly used and can be regarded as the primary method in soft lithography. As a first step, proteins are incubated on an elastomeric stamp carrying microscale pattern elements, forming a thin monolayer on the surface of the stamp. The stamp is then placed face down onto a previously functionalized substrate. When the stamp is removed, a pattern of protein is transferred to the substrate (Fig. 2A). Subsequent incubation of the biomolecules allows selective binding of proteins. The binding affinity of incubated proteins to the printed biomolecules defines whether they bind to structures or to the functionalized spaces between them. Additionally, further protein immobilization steps can be included using proteins with high binding affinity to the immobilized proteins [72].

Recent modifications in substrate material, surface functionalization, stamp material, and the printing structure have advanced μCP and broadened its applications [31,33,38,47,59]. Common substrates used in combination with this technique includes glass slides and cyclic olefin polymer (COP) foils. Due to the high flexibility of COP foils, large patterned areas of this substrate material need to be stabilized with 384-well plate casting for imaging purposes [54,55,63].

PDMS-based stamps remain as the most preferred stamp materials in μCP. They are easy to handle, compatible with most proteins and can create homogeneous patterns on substrate [46]. One of the most printed pattern structures used for μCP are grid patterns with round- or square-shaped holes repeated at defined micron-scale intervals. This structure is used in combination with fill-up approach to immobilize target biomolecules (Fig. 1A) [10,33,37]. Another common structure is direct printing of biomolecules in square shaped patterns, complementary approach to the grid structures [13,68,73]. Both approaches provide high-resolution patterns that minimize errors during the printing process and facilitate straightforward analysis.

The advantages of μCP, such as cost-efficiency, reliability, simplicity, and fast production of micron-sized structures, make it the preferred method for patterning proteins on surfaces [47]. The structures have high precision and stability, and are compatible with various subsequent microscopy-based characterization techniques [17,43,69].

### 2.2 Nanocontact printing (nCP)

Similar to μCP, in nCP an elastomeric stamp is used to deposit proteins onto the substrate. However, nCP offer higher control over patterning and allows for creating nanometer-scale structures (Fig. 2B) [13]. Typically, target proteins such as antibodies or ligands, are directly printed on the surface for better control over protein deposition and consequently, for achieving higher resolution [68,70,71,73,[76], [77], [78]]. Alternatively, antigens can be printed on the substrate and their interaction with primary and secondary antibodies can be investigated. This approach can be used to study antigen-antibody interactions with subsequent cell incubation [79].

PDMS is the most used material for stamp fabrication in nCP. The choice of printed structure is selected based on research purposes. For instance, line structures produce multifunctional surfaces, arranging intersecting lines to create complex patterns [71,78]. On the other hand, dot patterns, down to sub-100 nm patterns, facilitating biomolecule and cell interaction observation [68,73,80]. By combining different dot sized patterns, each representing distinct protein type, multifunctional patterned surfaces can be produced [70].

Similar to μCP, nCP also provides the possibility to deposit fluid-supported lipid bilayer patterns on substrate. Fibronectin can be used as anchor point for cell behaviour and interaction studies [81,82].

nCP is a cost-effective and precise printing method, enabling the fabrication of nanometer-resolution patterns [29]. It has contributed to the study of cell biology, including adhesion dynamics, immune cell interactions, membrane protein organization, and calcium flux measurement in both fixed and live cells [1,23,79]. However, it has been demonstrated that direct printing of biomolecules may compromise the functionality of delicate proteins due to the pressure and stress exerted during the printing process [11]. Additionally, the random orientation of proteins during deposition may block the interaction site and reduce interaction efficiency [19]. To overcome this issue, biomolecules such as streptavidin can be directly printed on the substrate, followed by the incubation of biotinylated proteins. This approach ensures the better orientation of the biomolecules for interaction [30,83].

### 2.3 Capillary nanostamping

Capillary nanostamping uses a non-elastomeric stamp to create submicron to nanometer patterns over large-scale areas using capillary force. Unlike elastomeric stamps, the stamp used in this technique can be made from materials like block copolymers [14] or spongy mesoporous silica [51]. These stamps are loaded with ink or biomolecules which is subsequently transferred to the substrate through capillary movement (Fig. 2C). The size and resolution of printed structures can be adjusted by varying contact time and pressure applied during printing. Chemical compounds (e.g. 1-dodecanethiol, heterocyclic silanes) or biomolecules (e.g. BSA, rhodamine B) have been printed on pre-functionalized surfaces using this technique [14,50,51,84].

This technique delivers higher resolution, and more flexibility in pattern design [50]. Furthermore, the possibility to use diverse stamp materials allows for better control over the influence of stamp properties on pattern quality. However, capillary nanostamping requires precise optimization of stamp, ink, and surface properties to ensure the reproducibility of the patterns. Additionally, the fabrication of mesoporous stamps can be time-consuming which may limit its application.

### 2.4 Polymer pen lithography (PPL)

PPL combines the precision of Dip-Pen Nanolithography (DPN) with the scalability of μCP [16]. In DPN, a sharp cantilever tip is used for scanning and deposition of biomolecules on the surface. The tip is inserted into the ink or protein solution and loaded on the high-resolution scanning probe. The ink is positioned on the substrate exploiting the meniscus between the ink on the cantilever tip and the surface. This technique enables the production of high-resolution and arbitrary patterns on the substrate [34]. However, patterning large areas is time-consuming when using a single tip and the use of cantilever arrays can become costly. To enhance the appeal of this high-resolution technique, numerous derivatives have been developed. In PPL, the sharp cantilever tip is replaced with a large elastomeric stamp, stabilized by a rigid backbone (usually a glass slide). The stamp can contain up to 11 million pyramid-shaped features, functioning as printing tips (Fig. 2D). The PDMS stamp is controlled by a high-precision piezo scanning setup, allowing for better control over contact time and pressure [66]. By varying these parameters, the size and shape of printed structures is adjustable, ranging in size from sub-100 nm to 10 μm and in shape from dot to square [85].

By switching to a deformative PDMS tip, PPL provides the ability to directly print diverse biomolecules, including phospholipids [53,75,86], oligonucleotides [87,88], antigens [18,89] and ligands [90] onto the substrate. Pre-functionalization of the surface is often required to allow specific protein deposition and to minimize unspecific binding. This can be achieved through the direct printing of surface activating materials, such as 16-Mercaptohexadecanoic acid (MHA) [15,66,85,90] and alkyne groups [27], or by coating the surface with activating materials such as NHS [89] to improve specific binding.

Beam Pen Lithography (BPL) is an interesting derivative of PPL, where UV light is passed through apertures implemented at the centre of PDMS pyramids and create polymerised patterns on photoreactive substrates [67]. This technique can further expand the application of PPL in creating high resolution structures.

In comparison to the previously described approaches, PPL provides better resolution and a broader range of patterns, with no restrictions on the distance between the structures. This method has been employed in the fabrication of structures in microfluidic systems [18,87] and creating DNA-based platforms for capturing oligonucleotides [45]. Furthermore, it is possible to simultaneously print more than one biomolecule on the substrate [75,88,89]. Moreover, by slightly tilting the stamp, printing size gradient structures on the substrate can be achieved [53]. However, this setup is more complex and expensive, as it requires high precision scanning device. In addition, the efficiency of ink transfer can be affected by various factors such as ink composition, surface tension, tip structure, and environmental parameters such as humidity and temperature. These variables are required to be carefully optimized to ensure reliable results. Moreover, the precise control of the setup requires skilled operators and calibrated equipment which can also be noted as a limiting factor.

## 3 Biomolecule toolbox for protein patterning

**Fig. 3:**
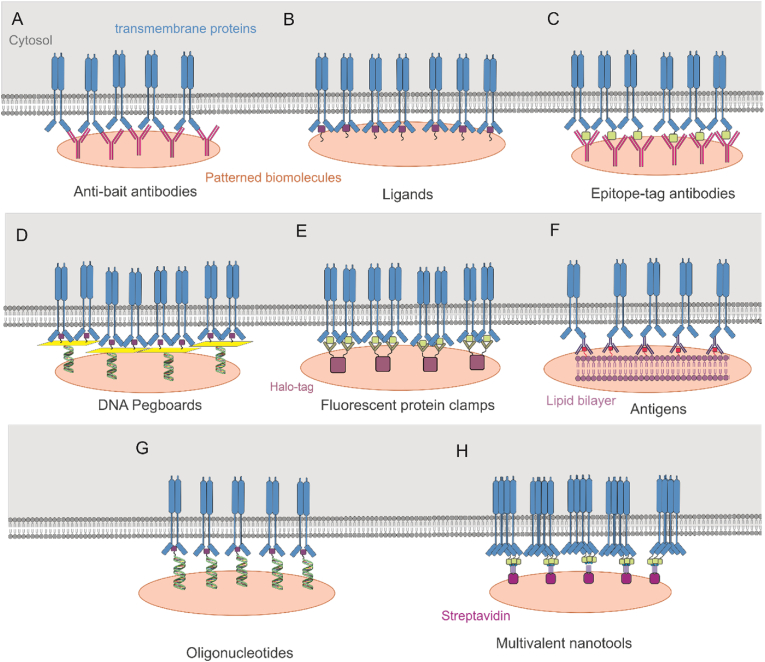
Schematic presentation of key strategies employed in the biomolecule toolbox. A) anti-bait antibodies are used to directly interact with transmembrane proteins, B) immobilized ligands interact with receptors induce activation, C) epitope-tag antibodies immobilize proteins tagged with specific proteins, D) DNA pegboards are used to immobilize transmembrane proteins on the substrate with specific spacing, E) Fluorescent protein clamps are immobilized on the substrate using their Halo-tag portion and interact with GFP-tagged membrane proteins, F) antigens located on lipid bilayers improve the interaction efficiency with antibodies, providing optimum antibody orientation for interacting with cell membrane proteins, G) oligonucleotides interact specifically with their complementary ssDNA, improving the efficacy of bait protein immobilization, and H) multivalent nanotools interact with multiple proteins simultaneously, inducing receptor activation and clustering. Each method provides a unique application for detailed PPI analysis resolution ranging from μm to nm.

One of the key applications of protein patterning is to create a controlled environment for subcellular PPI studies. The enrichment of cell membrane proteins in patterned surfaces allows for targeted probing of cellular processes [1]. In this context, cellular interactions play crucial role, and in this review, we focus on how protein patterning can provide a high resolution and adaptable environment for studying such interactions. The following section provides an overview on the available biomolecule toolbox for membrane-anchored protein patterning (thereafter defined as “bait” or “bait protein”), including the most commonly used biofunctional protein patterns such as anti-bait antibodies, ligands, epitope-tag antibodies, DNA pegboards, protein clamps, antigens, oligonucleotides, and multivalent nanotools (Fig. 3).

### 3.1 Anti-bait antibodies

Anti-bait antibodies allow specific biomolecule immobilization by interacting with their respective epitopes (Fig. 3A) [35,57,58]. These antibodies, when conjugated with affinity tags or functional groups, can enrich bait protein at the cellular membrane. This approach is employed for immobilization of high-affinity proteins and transmembrane receptors onto patterned areas [73]. Additionally, real-time measurement of receptor activation through in-situ ligand addition stays possible [57].

### 3.2 Ligands

Ligands and small molecules with specific binding properties, is used for immobilizing high affinity binding proteins on patterned surfaces [73,76,77] (Fig. 3B). Several factors can affect the interaction efficacy such as chemical modification of the surface [68], type, density and orientation of the immobilized ligands [45,91]. Membrane protein interactions with immobilized ligands have been used for studying interaction affinity, protein functionality, receptor activation and signalling [45,84,91].

### 3.3 Epitope-tag antibodies

Epitope-tag antibodies immobilize proteins tagged with specific epitopes in the cell membrane onto patterned surfaces (Fig. 3C) [23,55,76]. Tags like hemagglutinin (HA) can be used to investigate receptor-ligand interactions, dynamics of membrane protein, and signal transduction pathways [76]. Proteins tagged with green fluorescent protein (GFP) or mCherry can be captured using antibodies against their specific fluorescent tags [30,49,83]. Short peptide tags such as Halo-tags can be used for the capture of bait proteins using anti-Halo nanobodies [78]. Additionally, His- and FLAG-tags can be used for creating recognition sites for purification, immobilization and interaction studies [3,78,84,92]. Variety of protein tags are available which can improve the flexibility of experiments according to specificity and efficiency needed for PPI measurements.

### 3.4 DNA pegboards

DNA pegboards use DNA strands for positioning proteins in nano-scale distances onto micropatterned surfaces (Fig. 3D). In this system, a long DNA stand is used to create customized pegboard structures. These pegboards interact with complementary immobilized DNA strands in the pattern elements. By positioning interacting proteins, such as ligands, on top of these pegboards, nanoscale resolution bait protein enrichment can be achieved [45,91]. DNA pegboards are adaptable for various biomolecules, though complex design and preparation steps can be time-consuming and expensive [93].

### 3.5 Fluorescent protein clamps

Fluorescent protein clamps are specifically used for immobilizing GFP-tagged proteins on the substrate. They are originated from engineered ankyrin repeat proteins (DARPins) with high affinity for GFP [94]. Philippi et al. have advanced this system by integrating two GFP-clamps to a Halo-tag to create a dual function system. In this system, the Halo-tagged BSA molecules are first covalently attached to the patterned substrate. Then the modified GFP-clamps are added to the substrate to interact with the Halo-tagged BSA from the Halo-tag portion of the clamp. The GFP-tagged portion of the clamp are spatially oriented in the way to interact with GFP-tagged membrane proteins [84]. The design of such specific system requires careful optimization and preparation.

### 3.6 Antigens

Antigens are used to capture specific antibodies or proteins on the surface (Fig. 3F) [46]. When they are integrated into lipid vesicles and form bilayer-patterned surfaces, selective immobilization of specific antibodies or proteins in patterned areas is achieved and bait transmembrane protein enrichment is facilitated [35,77,86]. This setup allows for analysing antibody binding affinities, cellular responses, and the detection of specific antibodies in diagnostic applications [76]. Antigen-antibody interactions are highly specific and are adaptable to the diverse experimental conditions.

### 3.7 Oligonucleotides

Oligonucleotides provide a customizable strategy for specific immobilization of biomolecules (Fig. 3G) [1,10,72]. They can be conjugated to biomolecules such as ligands or antibodies and be immobilized on the patterned surfaces to interact with bait proteins in cell membranes. The immobilization happens through the complementary interaction of ssDNA conjugated biomolecule with immobilized complementary ssDNA on the surface [3,87,88,95].

Oligonucleotides bind precisely to complementary sequences, increasing the specificity of interactions. In addition, it is adaptable to various biomolecules since oligonucleotides can conjugate with different proteins and peptides. However, conjugating proteins with oligonucleotides can be challenging due to the linking difficulties. Moreover, proteins can lose their functionality during conjugation and the oligonucleotide attachment can interfere with the natural activity of the protein or blocks its binding sites [72].

### 3.8 Multivalent nanotools

Multivalent nanotools are nanoscale constructs designed to enhance functionality by engaging in multiple ligand-receptor interactions simultaneously (Fig. 3H). By placing multiple binding sites on a single scaffold, the binding affinity and specificity is improved compared to monovalent interactions. This improvement is essential in investigating complex biological systems, where interactions are weak or transient [24,96]. Examples of multivalent nanotools include DNA origami structures and protein-based scaffolds like streptavidin-biotin systems [24].

In protein patterning, the central scaffold, such as trivalent N-nitrilotriacetic acid (trisNTA) nanotool, can be functionalized with ligands. TrisNTA consists of three NTA (nitrilotriacetic acid) groups attached to a central scaffold. These nanotools can target tagged membrane proteins such as His6-tagged with high specificity [48,97]. A nanotool with multiple ligands can bind multiple receptor domains and cause ligand-induced or -free receptor activation and clustering [96]. However, designing and synthesizing multivalent nanotools requires careful consideration of the scaffold choice, ligand density, spatial arrangement, and overall stability [24].

The biomolecule toolbox for protein patterning offers variety of methods which are adaptable to different research purposes. Optimizing and advancing these approaches will open new possibilities in protein patterning and cell membrane studies. However, the potential for pre-activation of immobilized receptors on patterned surfaces should be considered. In this phenomenon, surface interactions may induce conformational changes in the proteins that can lead to receptor dimerization or clustering even in the absence of ligand binding. This can affect experimental results by mimicking false-positive activation signals [23]. Addressing the advantages and limitations of each immobilization strategy can assist in selecting the suitable technique and expand the application of protein patterning in PPI analysis.

## 4 Protein deposition strategies

**Fig. 4:**
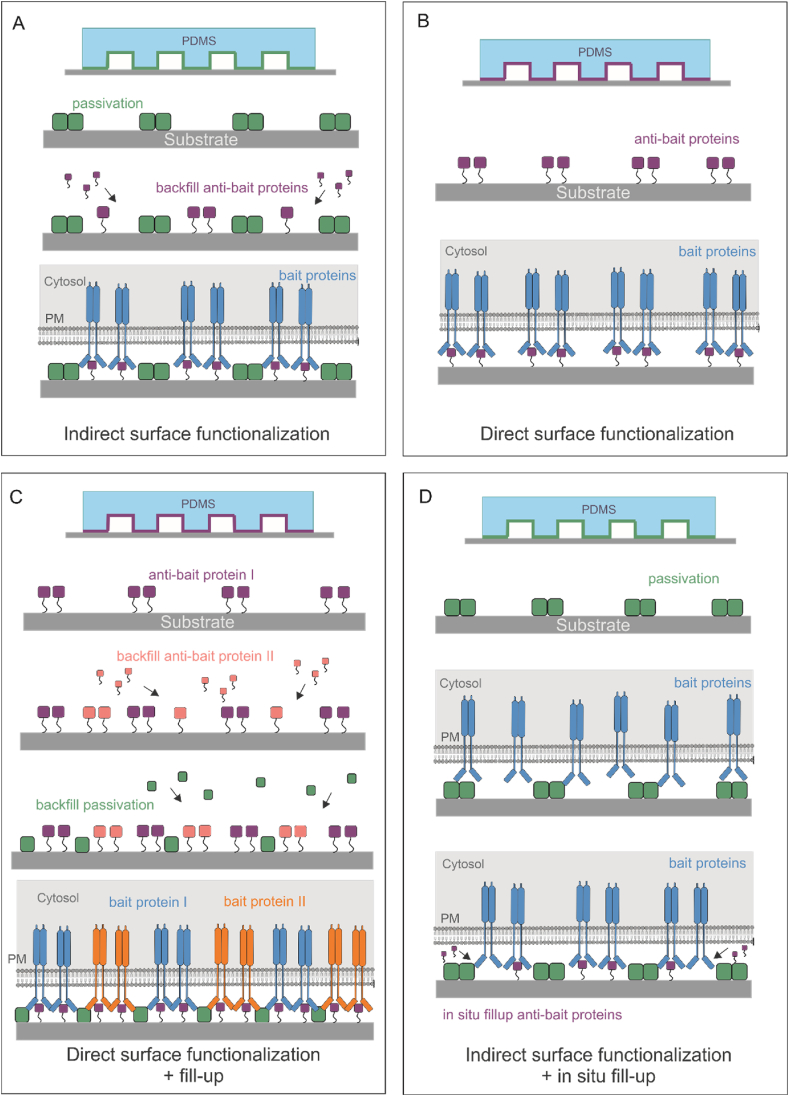
Schematic presentation of protein deposition strategies. A) Passivating structure is directly printed on the substrate. This is followed by immobilization of anti-bait protein in between the printed structure. Transmembrane bait proteins accumulate in the patterned areas and interact with the immobilized proteins. B) Anti-bait proteins are printed directly onto the substrate. Transmembrane bait proteins interact with the printed proteins. C) Similar to B, anti-bait proteins (anti-bait protein I) are directly printed on the substrate. This is followed by addition of second anti-bait (anti-bait protein II) and passivating proteins to bind in between the printed proteins and passivate the empty spaces respectively. Transmembrane bait proteins interact with both immobilized anti-bait proteins. D) Similar to A, passivation structure is directly printed on the substrate. This is followed by cell attachment to the substrate. afterwards, anti-bait proteins are added to the substrate. These proteins penetrate the gap between the cell and substrate and bind to empty patterns in between the passivating structures. The process is tracked by observing the accumulation and interaction of transmembrane bait proteins in the patterned areas.

Anti-bait protein deposition strategies on patterned surfaces provide a variety of methods to achieve precise control of protein immobilization and spatial arrangement. In order to achieve such high precision systems, several deposition strategies have been introduced [23,35]. The following section will discuss four main strategies: indirect functionalization, direct functionalization, direct functionalization followed by fill-up, and indirect functionalization followed by in-situ fill-up (Fig. 4).

### 4.1 Indirect surface functionalization

Indirect functionalization involves direct printing of passivating structure on substrates, followed by subsequent binding of biomolecules to the unpassivated pattern elements (Fig. 4A) [65]. This deposition strategy is often employed in combination with μCP. Through the specific interactions and the controlled presentation of proteins to cells, a multitude of cell behavioural parameters may be studied, including cell adhesion, spreading, and the activation of certain signalling pathways [17,55].

### 4.2 Direct surface functionalization

In the direct functionalization approach, as frequently used in nCP (but not limited to this approach), anti-bait proteins are directly printed and immobilized on the surface (Fig. 4B) [73,76]. This technique is a one-step method and as opposed to indirect functionalization, the passivation step is optional [73]. However, direct printing may affect the orientation and functionality of the immobilized proteins. The direct functionalization method is one of the simplest patterning methods which enables precise manipulation of cell behaviour and functions [64,78].

### 4.3 Direct surface functionalization with subsequent fill-up

In this method, the anti-bait protein I is directly printed on the substrate. Subsequently, the anti-bait proteins II are added to the substrate, binding to the empty spaces between the printed structure. At this stage, a passivating solution is added to the surface to fill-up empty spaces to avoid non-specific binding (Fig. 4C) [28,33]. This deposition method increases the specificity of protein immobilization, minimizes background signals and improves the cell adhesion to the surface.

### 4.4 Indirect surface functionalization with subsequent fill-up

In this strategy, the passivating protein is first printed onto the surface. After the cells are adhered to the patterned substrate, anti-bait proteins, such as ligands, are added to the substrate. They will penetrate into the empty gap between the cells and substrate and bind to the empty spaces in between the printed structures (Fig. 4D). This method combines the specificity of indirect functionalization with the ability for in-situ stimulation of PPIs in live cells [48,78,98]. However, this method is limited to small biomolecule as they need to penetrate beneath the attached cells on the surface.

## 5 Soft lithography-based interfaces for subcellular protein interaction analysis

Subcellular PPI analysis has been used to study protein complexes and to identify their roles in cellular responses. Various techniques like co-immunoprecipitation (co-IP), affinity purification, yeast two-hybrid assays, two-hybrid antibody assays and mass spectrometry have been used to investigate the structure of these complexes [58,76,92,99]. Soft lithography-based approaches and microfluidic tools have improved the efficiency and precision of traditional methods and can be combined with real-time quantitative analysis methods [32,33]. These dynamic analysis provide information on various physiological functions such as understanding the role of specific receptor regions on its activation properties, the effect of ligands or inhibitors on receptor activation or inhibition, and the role of specific stimuli on protein internalization [55,92,99].

Anti-bait patterned surfaces simulate natural cellular environment with high specificity and control over protein interactions [35]. However, squeezing high number of membrane proteins into small spaces affects their natural conformation and functionality. Additionally, it can cause pre-activation, leading to partial activation, dimerization, or clustering [57], Furthermore, or endocytosis frustration which can interfere with their natural behavior [45,100].

**Fig. 5:**
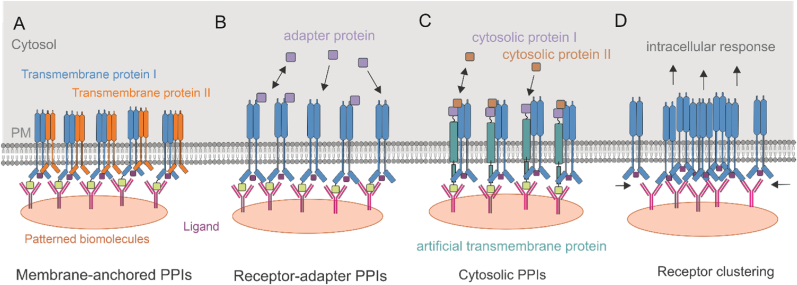
Schematic presentation of PPI analysis readout modalities. A) epitope tagged transmembrane receptors are immobilized on the substrate. When activated, they form homo- or heterodimer with interacting partners across transmembrane. B) the activation of immobilized membrane protein leads to interaction with adapter protein in cytosol and activating related signalling pathways C) the immobilized artificial transmembrane protein on the substrate makes the study of cytosolic proteins interactions on micropatterned surfaces possible. D) cluster formation of immobilized transmembrane receptors on micropatterned surfaces can assist in investigating their role on cellular signalling.

This chapter summarizes important studies investigating subcellular PPI analysis using soft lithography-based contact printing (Fig. 5).

### 5.1 Membrane-anchored PPIs

Membrane proteins forms homo- and heterodimers upon activation (Fig. 5A). Studying these interactions provide valuable insight into cell adhesion, receptor activation and cell signalling [68].

We explored the heterodimerization of glucose-dependent insulinotropic polypeptide receptor (GIPR) and glucagon-like peptide-1 receptor (GLP-1R) [49]. Both receptors are present on pancreatic β-cells, indicating a potential for heterodimer formation. By utilizing μCP, FRET, and bioluminescence resonance energy transfer (BRET) saturation experiments, it was confirmed that GLP-1R and GIPR form heterodimers.

Sevcsik et al. investigated the distribution of glycosylphosphatidylinositol (GPI)-anchored proteins on micropatterned surfaces [83]. These proteins lack significant cellular functions and minimal ectodomain interactions, making them ideal for investigating their behavior within lipid-ordered domains in live cells. Their findings indicated that GPI-anchored proteins behave as obstacles, limiting the diffusion of other membrane components without affecting their environment.

### 5.2 Receptor-adapter PPIs

Receptor-adapter PPIs are crucial for connecting the activation of cell surface receptors to intracellular signalling pathways (Fig. 5B). Receptors recruit adapter proteins, initiating downstream signalling cascades [78].

Schwarzenbacher et al. were among the first to use soft lithography for analysing subcellular PPIs. They developed micropatterning techniques to study interactions between CD4 and Lck in living cells, including mutations [20]. The authors discovered that CD4 interact with 9 Lck molecules on average.

We investigated the quantitative activation of epidermal growth factor receptor (EGFR) using micro-patterned surfaces [57]. Our aim was to elucidate the effect of the ligand (EGF) and clinically tested inhibitors on the interaction of EGFR with growth factor receptor-bound protein 2 (Grb2). We found that Grb2 recruitment to EGFR depended on EGF presence, which was significantly blocked by EGFR inhibitors.

In another study, the effect of clathrin-mediated endocytosis on protein interactions was investigated [45]. It was observed that clathrin create internalization hotspots within the patterned areas, causing non-specific co-recruitment of bait and prey proteins. These hotspots pose challenges in accurately interpreting the acquired data.

We investigated DNA origami-based molecular rulers to explore how nanoscale ligand spacing impacts membrane receptor activation and signal transduction. EGFR was selected for this purpose and its interaction with Grb2 was analysed [91]. We found that maximum EGFR activation is achieved when EGF ligands are placed approximately 30–40 nm apart.

Motsch et al. focused on quantitative analysis of the interaction between T cell receptor (TCR) and Syk kinase ZAP70 [80]. These interactions are crucial for T cell activation and immune responses. Using fluorescence recovery after photobleaching (FRAP), the authors were able to report quantitative data of TCR-ZAP70 interaction.

Wedeking et al. developed a tool consisting of an anti-GFP nanobody and Halo-Tag (NB-HaloTag) to immobilize type I interferon receptors (IFNAR1) on functional surfaces [78]. The substrate is patterned with Halo-Tag ligand. After cells are attached to the substrate, IFNAR1 are rearranged in patterned structure upon addition of NB-HaloTag. IFN signaling was investigated through dimerization of IFNAR1 and IFNAR2 and interaction with their adapter protein.

### 5.3 Cytosolic PPIs

Cytosolic interactions play important roles in various regulatory mechanisms including protein distribution, signal transduction, and cell cycle. Anti-bait-patterned surfaces are used in combination with artificial transmembrane proteins to investigate cytosolic PPIs (Fig. 5C).

Gandor et al. initiate the study of cytosolic PPIs by developing the bait-presenting artificial receptor (bait-PARCs) system [95]. This system was immobilized on the surface using oligonucleotides to create micropattern antibody arrays. In this study, G protein-coupled receptors (GPCR) was used to validate its biological application. The bait-PARC system demonstrated the ability to measure multiprotein interaction kinetics with high dynamic range and sensitivity.

We adapted Gandor's bait-PARC system to study Grb2-mediated signalling using an artificial transmembrane bait construct and microstructured antibody arrays on substrates [55]. The primary objective was to investigate proteins involve in EGFR signalling. We identified protein complexes involved in EGFR signalling, such as Grb2:SOS1 and Grb2:Gab1, as well as transient agonist-dependent interactions (Grb2:Shc1 and Grb2:PI3K). Our analysis revealed differences in stability and exchange kinetics of these interactions.

### 5.4 Receptor clustering

Receptor clustering refers to the accumulation of cell surface receptors leading to amplification and cellular signalling (Fig. 5D).

Diez-Ahedo et al. investigated the dynamic rearrangement of integrin nanoclusters on ligand immobilized surfaces [68]. Integrins were selected due to their critical role in various cellular processes including interactions, proliferation, and survival. In the study it was showed that integrin nanoclusters dynamically reorganize in response to the spatial distribution of ligands.

Dirscherl et al. explored protein clusters in major histocompatibility complex (MHC) I proteins using a two-hybrid micropattern assay involving antibody micropatterns [76]. This assay supports investigating interactions and conformational changes of MHC I proteins. Their results showed that in-cis interactions are limited to MHC I heavy chains, leading to dissociation of light chains from the complex.

We developed a tool to investigate the activation of neuropeptide Y2 hormone receptors (Y2R) [98]. It was demonstrated that the clustering of Y2R receptors facilitated dynamic exchange of histidine-tagged Y2R. This leads to increased cytosolic calcium level and enhanced cell migration in the absence of ligand. Pre-clustered Y2R cells showed higher responses to ligand-induced activation.

In a recent study, we developed a nanotool to investigate TCR clustering and signalling [96]. Engineered conjugation pair and peptide-loaded MHC (pMHC) molecules were used to compare ligand (pMHC)-induced and ligand-independent receptor clustering. The nanotools were equipped with streptavidin binding peptide, allowing for the immobilization on the streptavidin patterned surfaces. The results indicated pMHC-induced clustering was denser and more dynamic than ligand-free clustering.

Philippi et al. investigated the mechanisms behind the formation and function of Wnt signaling pathway [84]. With the help of biofunctional nanodot arrays (bNDAs), authors aimed to understand the role of receptor clustering and liquid-liquid phase separation (LLPS) in activation of co-receptor Lrp6 and Frizzled-8. Additionally, significant co-recruitment of Frizzled-8 with Axin-1 and Disheveled-2 to Lrp6 nanodots, even without ligand stimulation was depicted.

Meddens et al. investigated the interaction between CD6 and TCR-CD3 [77]. T cell activation leads to the interaction at the centre of immunological synapse (IS). The results indicated the microcluster formation of CD6 with TCR/CD3 on both supported lipid bilayers and antibody-patterned surfaces.

Shen et al. studied the spatial organization of CD28 clusters relative to CD3 and its role on T cell activation and IL-2 production [70]. The results indicated that when the anti-CD3 antibodies are surrounded by anti-CD28, the IL-2 production in CD4^+^ T cells is increased which enhances the NF-κB translocation and PKB/Akt signalling.

Witsenburg et al. explored the effect of TCR and CD28 signalling, a costimulatory receptor, on microcluster formation in T cells [71]. The study showed that CD28 co-stimulation increases microclusters signalling and promotes cell spreading. However, Src homology-2 protein (SHP2) reduced phosphorylation levels in tyrosine phosphorylation of clusters.

## 6 Conclusion and future perspective

Soft lithography-based micro- and nanocontact printing strategies are invaluable for biomolecule patterning and PPI studies. These techniques have several advantages, including controlled biomolecule deposition, high degree of biocompatibility, and ease of implementation [3,23]. High resolution protein patterning on substrates facilitates the study of protein interactions, signalling cascades, and cellular processes [35]. Furthermore, soft lithography-based approaches offer a variety of deposition techniques for the fabrication of complex and multifunctional structures [19].

Future advancements in this approached will enhance the resolution and accuracy of protein patterning and studying dynamic interactions. These advancements include modifications in stamp design, surface activation, and imaging technologies, which are critical for improving both the spatial and temporal resolution of PPI studies [36]. By designing stamps using stimuli-responsive materials, greater control over biomolecule deposition can be achieved. Additionally, innovative substrate functionalization methods can reduce the preparation time and increase the precision and reproducibility.

Combining soft lithography with advanced analytical techniques (e.g. mass spectrometry) and new technologies can develop multifunctional platforms [2,101]. Moreover, the potential for 3D printing using soft lithography can further enhance the study of protein interactions by creating more physiologically relevant models [102,103]. In addition, the study of PPI in biological environment can be facilitated by placing a thin slice of biological sample on collagen micro patterned substrates or by patterning collagen onto the biological sample [104,105]. In addition, combining these approaches with patterned lipid bilayers improves the study of transmembrane proteins and complex cellular processes [20].

Furthermore, integrating with translational approaches can fill the gap between early-stage discoveries and real-world applications such as drug discovery processes and identifying lead compounds or optimizing hit-to-lead (H2L). These assays can be used to study the effect of lead compounds on cell behaviours such as proliferation, differentiation and migration. Additionally, such patterned substrates can be designed to investigate different molecular interactions at subcellular level. When targeting extracellular interactions, patterned proteins such as antibodies or antigens can be used to immobilize transmembrane receptors, as drug targets, to investigate interaction, activation and binding affinity of possible drug compounds and to measure the selectivity and efficacy of the selected targets. This strategy can provide insight into quantitative evaluation of binding kinetics and the effect of these compounds on downstream signalling pathways. Studying intramembrane interactions on patterned surfaces is possible when implementing patterned lipid bilayers or membrane-bound proteins. Small molecules, peptides and antibodies can inhibit, enhance or regulate these interactions by inducing receptor clustering, dimerization and drug-related conformational changes. Intracellular interactions such as cytosolic PPIs, enzyme activity and the dynamics of uptake and distribution of drug targets can be investigated using patterned substrates. For example, artificial transmembrane proteins can assist in modifying cytosolic PPIs to investigate and adjust the binding affinity, competition kinetics and post-translational modifications of drug compounds. They can facilitate the understanding of drug efficacy and resistance by studying intracellular clustering, kinase-substrate interactions and downstream effector recruitment. Furthermore, patterned surfaces can be employed to get insight into the effect of spatial organization on the uptake and distribution of intracellular transport of drug-targeted molecules.

By printing patterns over large areas, soft lithography patterning techniques can be applied to medium-throughput screening (MTS), capable of testing up to 20–50K compounds, as well as high-throughput screening (HTS) systems for larger scale assays [17,55,66,106]. The achievable throughput depends on the choice between using micropatterned chips or 384-well plates [107]. Furthermore, higher efficiency in measurement can be achieved when integrating such systems with microfluidic systems. Further advancements in the use of micropatterned surfaces for drug screening could revolutionize the identification of lead compounds and prediction of bio-efficacy.

One key factor of studying biomolecule interactions on patterned surfaces is to establish the 1:1:1 interaction interface [108]. Several factors can affect the efficiency of this type of interactions on patterned surfaces. The concentration of deposited biomolecules on the surface determines the number of molecules available for interaction. However, high concentration of the immobilized biomolecules can increase the number of non-specific binding of molecules. The deposited proteins should have low dissociation constant (K_d_) for interacting proteins to achieve improved interaction stability and efficiency. By considering these factors and finding the balance between the protein concentration and binding affinity, such patterned surfaces can be a practical tool for competition-based primary screens and measuring accurate kinetic measurements and screening performances.

In summary, soft lithography-based micro- and nanocontact printing techniques have a great potential to enhance PPI research and current protein deposition strategies. Furthermore, the application of these approaches in drug discovery to address involved challenges including protein pre-activation and 2D-3D structural differences, will open new paths for studying disease mechanisms and the development of new therapies.

## CRediT authorship contribution statement

**Tina Karimian:** Writing – review & editing, Writing – original draft, Visualization, Methodology, Conceptualization. **Peter Lanzerstorfer:** Writing – review & editing. **Julian Weghuber:** Writing – review & editing, Project administration, Funding acquisition, Conceptualization.

## Funding

This research was funded by the province of Upper Austria as part of the University of Applied Sciences Upper Austria Center of Excellence for Technological Innovation in Medicine (TIMed CENTER); the 10.13039/501100006012Christian Doppler Forschungsgesellschaft (Josef Ressel Center for Phytogenic Drug Research); the 10.13039/501100002428Austrian Science Fund (10.13039/501100002428FWF) [project I4972−B]; and the ‘Dissertationsprogramm der Fachhochschule OO 2020′ with the financial support of the province of Upper Austria (10.13039/501100004955Austrian Research Promotion Agency (10.13039/501100004955FFG) [grant number #881300].

**Table:** 

CP method	Advantages	Disadvantages	Substrate chemistry	Biomolecule patterns	Ref.
μCP	- High resolution	- Limited to planar substrates	- unfunctionalized and Functionalized glass with Epoxy, metal-ion coating or streptavidin)	- Direct printing proteins such as streptavidin, ligands, antibody or lipid bilayers	[10,23,43,46,55,57,58,65]
	- Compatible with various substrate materials	- Reduce in quality of stamp over time	- Cyclic olefin polymer foils	- Passivating biomolecules: include BSA, Pll-g-PEG or Fibronectin.	
	- Simple and cost-effective	- Precise stamp alignment is necessary	- Gold-coated substrate		
	- Fast pattern production	- Potential for contamination	- Silicon substrates		
	- Reusable stamp	- limited to space in between the structures			
	- No specific and expensive equipment is required				
	- Compatible with functionalized SAMs				
nCP	- High resolution patterning (nm range)	- Loss of function of printed proteins due to pressure and stress applied during printing	- Functionalized glass substrate such as Epoxy-coated	- Direct printing of proteins such as oligonucleotides, lipids, antibodies or streptavidin	[10,27,29,43,[68], [69], [70],^73^,^75^,^81^]
	- Cheap, practical, easy, and fast method	- Random orientation of proteins during direct printing	- Untreated glass slide	- Passivating materials including fibronectin, Pll-g-PEG or BSA	
	- Reusable stamps	-Limited to space in between structures	- Silicon substrate		
	- no specific equipment is required				
Capillary nanostamping	- Versatility in stamp materials (e.g., block copolymers, silane, spongy mesoporous silica).	- Requires careful optimization of stamp and ink properties for each specific application.	- Gold and titanium coated glass	- Functionalization reagents or -Proteins such as BSA, silane-based solutions, or rhodamine B.	[14,50,51,84]
	- Enhanced control over micro-stamp creation and intricate patterns.	- - - Complex stamp fabrication Timeconsuming.	- Unfunctionalized glass substrate	- Aminosilane nanodots	
	- printing several times without the need for reinking the stamp	- Limited to certain ink materials and applications, depending on stamp characteristics.	- Hydroxyl-terminated glass substrate		
	- Possibility to create size gradient structures on the substrate by varying contact time and pressure.				
PPL	- Higher resolution and variety of patterns compared to μCP	- Requires more complicated and expensive setup compared to conventional μCP	- Gold substrate functionalized with MHA	- Direct printing of various biomolecules such as phospholipids, oligonucleotides, or antigens.	[15,16,18,19,53,75,86,88,89]
	- Not limited to spaces in between structures	- Time consuming system adjusting process	- Unfunctionalized glass substrate		
	- Produce multifunctional surfaces within single printing		- Functionalized glass or silicon, with alkyne−azide, NHS, fibronectin, BSA, hydrophobic Bis-epoxypolyethylenglycol or poly-bisphenol-A-glycidyl polymer		
	- Printing process is fast and simple				
	- Can be used on wide variety of substrate material				
	- Possibility to create size gradient structures on the substrate in single printing step.				

## Declaration of competing interest

The authors declare that they have no known competing financial interests or personal relationships that could have appeared to influence the work reported in this paper.

## Data Availability

Data will be made available on request.

## References

[1] Blättler T., Huwiler C., Ochsner M., Städler B., Solak H., Vörös J., Grandin H.M. (2006). J. Nanosci. Nanotechnol..

[2] Dias A.D., Kingsley D.M., Corr D.T. (2014). Biosensors.

[3] Humenik M., Winkler A., Scheibel T. (2021). Biopolymers.

[4] Lenci a S., Tedeschi b L., Pieri a F. (2011). C. Domenici b.

[5] Pardo Laura, Cris Wilson W. (2003). Thomas Boland.

[6] Nicolau Dan V., Ivanova Elena P., Fulga Florin, Filipponi Luisa, Viezzoli Andrea, Dobroiu Serban, Alekseeva Yulia V., Duy K. (2010). Pham.

[7] Lee KB, Park SJ, Mirkin CA, Smith JC, Mrksich M. 295 (2002) 1702–1705. 10.1126/science.1067172 11834780

[8] Kane R. (1999). Biomaterials.

[9] Xia Younan, George M. (1998). Whitesides.

[10] Alom Ruiz S., Chen C.S. (2007). Soft Matter.

[11] Juste-Dolz A., Avella-Oliver M., Puchades R., Maquieira A. (2018).

[12] Milan Mrksich, George M. (1995). Whitesides.

[13] Schütz G.J., Weghuber J., Lanzerstorfer P., Sevcsik E. (2017). Methods Mol. Biol..

[14] Guo L., Philippi M., Steinhart M. (2018). Small.

[15] Eichelsdoerfer D.J., Liao X., Cabezas M.D., Morris W., Radha B., Brown K.A., Giam L.R. (2013). Nat. Protoc..

[16] Voskuhl J., Brinkmann J., Jonkheijm P. (2014). Curr. Opin. Chem. Biol..

[17] Weghuber J., Sunzenauer S., Plochberger B., Brameshuber M., Haselgrübler T., Schütz G.J. (2010). Anal. Bioanal. Chem..

[18] Kumar R., Bonicelli A., Sekula-Neuner S., Cato A.C.B., Hirtz M., Fuchs H. (2016). Small.

[19] You C., Piehler J. (2016). Expet Opin. Drug Discov..

[20] Schwarzenbacher M., Kaltenbrunner M., Brameshuber M., Hesch C., Paster W., Weghuber J., Heise B. (2008). Nat. Methods.

[21] Dkhar D.S., Kumari R., Malode S.J., Shetti N.P. (2023).

[22] Alexandre-Franco MF, Kouider R, Kassir Al-Karany R, Cuerda-Correa EM, Al-Kassir A. 15 (2024). 10.3390/mi15091137 PMC11433824 39337797

[23] C. Dirscherl, S. Springer, Engineering in life sciences 18 (2018) 124–131. 10.1002/elsc.201700010 PMC6999577 32624894

[24] M. Florencia Sánchez 1 (2023).

[25] Bunde, K.A., Stamenović, D., Smith, M.L. J. Vis. Exp. (180) (2022) e63628. 10.3791/63628 35253805

[26] Zhang Kai, Han Xin, Li Ying, Yalan Li Sharon, Zu Youli, Wang Zhiqiang (2014). Lidong Qin.

[27] Bog U., de Los Santos Pereira A., Mueller S.L., Havenridge S., Parrillo V., Bruns M., Holmes A.E. (2017). ACS Appl. Mater. Interfaces.

[28] Coyle B.L., Baneyx F. (2016). Chemical communications (Cambridge, England).

[29] Coyer S.R., García A.J., Delamarche E. (2007). Angew. Chem..

[30] Fülöp G., Brameshuber M., Arnold A.M., Schütz G.J., Sevcsik E. (2018). Biomolecules.

[31] Desai R.A., Rodriguez N.M., Chen C.S. (2014). Methods Cell Biol..

[32] Sathish S., Ricoult S.G., Toda-Peters K., Shen A.Q. (2017). Analyst.

[33] Segerer F.J., Röttgermann P.J.F., Schuster S., Piera Alberola A., Zahler S., Rädler J.O. (2016). Biointerphases.

[34] Liu G., Hirtz M., Fuchs H., Zheng Z. (2019).

[35] Torres A.J., Wu M., Holowka D., Baird B. (2008). Annu. Rev. Biophys..

[36] Kaufmann T., Ravoo B.J. (2010). Polym. Chem..

[37] Jang M.J., Nam Y. (2015). Macromol. Biosci..

[38] Sadhu V.B., Perl A., Duan X., Reinhoudt D.N., Huskens J. (2009). Soft Matter.

[39] Zemła J, Szydlak R, Gajos K, Kozłowski Ł, Zieliński T, Luty M, Øvreeide IH, Prot VE, Stokke BT, Lekka M. 15 (2023) 51863–51875. 10.1021/acsami.3c09195 PMC10636731 37889219

[40] Foncy J., Estève A., Degache A., Colin C., Dollat X., Cau J.-C., Vieu C. (2018). PLoS One.

[41] Filipponi L., Livingston P., Kašpar O., Tokárová V., Nicolau D.V. (2016). Biomed. Microdevices.

[42] Abbasi AD, Hussain Z, Yang KL. 12 (2022).

[43] Lindner M., Tresztenyak A., Fülöp G., Jahr W., Prinz A., Prinz I., Danzl J.G. (2018). Front. Chem..

[44] Dubey R., Bhushan R. (2016). Bioanalysis.

[45] Lanzerstorfer P., Müller U., Gordiyenko K., Weghuber J., Niemeyer C.M. (2020). Biomolecules.

[46] Delamarche E., Niemeyer C.M., Mirkin C.A. (2004). Nanobiotechnology.

[47] Perl A., Reinhoudt D.N., Huskens J. (2009). Adv. Mater..

[48] Sánchez MF, Dietz MS, Müller U, Weghuber J, Gatterdam K, Wieneke R, Heilemann M, Lanzerstorfer P, Tampé R. 22 (2022). 10.1021/acs.nanolett.2c03506 PMC9614963 36219818

[49] Al-Zaid Bashaier, Chacko Siby, Ifeamalume Ezeamuzie Charles, Bünemann Moritz, Krasel Cornelius, Karimian Tina, Lanzerstorfer Peter (2022).

[50] Philippi M., You C., Richter C.P., Schmidt M., Thien J., Liße D., Wollschläger J. (2019). RSC Adv..

[51] Schmidt M., Philippi M., Münzner M., Stangl J.M., Wieczorek R., Harneit W., Müller-Buschbaum K. (2018). Adv. Funct. Mater..

[52] Clancy K.F.A., Dery S., Laforte V., Shetty P., Juncker D., Nicolau D.V. (2019). Biosens. Bioelectron..

[53] Kumar R., Urtizberea A., Ghosh S., Bog U., Rainer Q., Lenhert S., Fuchs H. (2017). Langmuir.

[54] Hager Roland, Forsich Christian, Duchoslav Jiri, Burgstaller Christoph, Stifter David, Weghuber Julian (2022). Peter Lanzerstorfer.

[55] Hager R., Müller U., Ollinger N., Weghuber J., Lanzerstorfer P. (2021). ACS Sens..

[56] Sunzenauer S., Zojer V., Brameshuber M., Tröls A., Weghuber J., Stockinger H., Schütz G.J. (2013).

[57] Lanzerstorfer P., Borgmann D., Schütz G., Winkler S.M., Höglinger O., Weghuber J. (2014). PLoS One.

[58] Lanzerstorfer P., Yoneyama Y., Hakuno F., Müller U., Höglinger O., Takahashi S.-I., Weghuber J. (2015). FEBS J..

[59] Ricoult S.G., Nezhad A.S., Knapp-Mohammady M., Kennedy T.E., Juncker D. (2014). Langmuir.

[60] Wang Z., Xia J., Luo S., Zhang P., Xiao Z., Liu T., Guan J. (2014). Langmuir.

[61] Wu H., Wu L., Zhou X., Liu B., Zheng B. (2018).

[62] Akarsu P, Grobe R, Nowaczyk J, Hartlieb M, Reinicke S, Böker A, Sperling M, Reifarth M. 3 (2021) 2420–2431. 10.1021/acsapm.1c00024 PMC8154209 34056615

[63] Hager R., Haselgrübler T., Haas S., Lipp A.-M. (2020).

[64] Jin M., Wu K., Wang M., Zhang Y., Yang C. (2023).

[65] Karimian T., Hager R., Karner A., Weghuber J., Lanzerstorfer P. (2022). Biosensors.

[66] Lee I.-N., Hosford J., Wang S., Hunt J.A., Curran J.M., Heath W.P., Wong L.S. (2018). J. Vis. Exp..

[67] X. Zhang, S. Ding, J. Magoline, A. Ivankin, C.A. Mirkin, Small (2022) e2105998. 10.1002/smll.202105998 35119205

[68] Diez-Ahedo R., Normanno D., Esteban O., Bakker G.-J., Figdor C.G., Cambi A., Garcia-Parajo M.F. (2009).

[69] Chalmeau J., Le Grimellec C., Sternick J., Vieu C. (2012). Colloids Surf. B Biointerfaces.

[70] Shen K., Thomas V.K., Dustin M.L., Kam L.C. (2008). Proc. Natl. Acad. Sci. U. S. A.

[71] Witsenburg J.J., Glauner H., Müller J.P., Groenewoud J.M.M., Roth G., Böhmer F.-D., Adjobo-Hermans M.J.W. (2013). PLoS One.

[72] Niemeyer C.M., Mirkin C.A. (2004). Nanobiotechnology.

[73] Dirscherl C., Palankar R., Delcea M., Kolesnikova T.A., Springer S. (2017). Small.

[74] MacNearney Donald, Mak Bernard, Ongo Grant, Kennedy Timothy E. (2016). David Juncker.

[75] Brinkmann F., Hirtz M., Greiner A.M., Weschenfelder M., Waterkotte B., Bastmeyer M., Fuchs H. (2013). Small.

[76] Dirscherl C., Hein Z., Ramnarayan V.R., Jacob-Dolan C., Springer S. (2018). Elife.

[77] Meddens M.B.M., Mennens S.F.B., Celikkol F.B., Te Riet J., Kanger J.S., Joosten B., Witsenburg J.J. (2018). Front. Immunol..

[78] Wedeking T., Löchte S., Birkholz O., Wallenstein A., Trahe J., Klingauf J., Piehler J. (2015). Small.

[79] Dirar Q., Russell T., Liu L., Ahn S., Dotti G., Aravamudhan S., Conforti L. (2020). PLoS One.

[80] Motsch V., Brameshuber M., Baumgart F., Schütz G.J., Sevcsik E. (2019). Sci. Rep..

[81] Kam L., Boxer S.G. (2001). J. Biomed. Mater. Res..

[82] Hovis J.S., Boxer S.G. (2001). Langmuir.

[83] Sevcsik E., Brameshuber M., Fölser M., Weghuber J., Honigmann A., Schütz G.J. (2015). Nat. Commun..

[84] Philippi M., Richter C.P., Kappen M., Watrinet I., Miao Y., Runge M., Jorde L. (2022).

[85] Huo F., Zheng Z., Zheng G., Giam L.R., Zhang H., Mirkin C.A. (2008). Science (New York, N.Y.).

[86] Angelin A., Bog U., Kumar R., Niemeyer C.M., Hirtz M. (2019). Polymers.

[87] Arrabito G., Schroeder H., Schröder K., Filips C., Marggraf U., Dopp C., Venkatachalapathy M. (2014). Small.

[88] Kumar R., Weigel S., Meyer R., Niemeyer C.M., Fuchs H., Hirtz M. (2016). Chemical communications (Cambridge, England).

[89] Zheng Z., Daniel W.L., Giam L.R., Huo F., Senesi A.J., Zheng G., Mirkin C.A. (2009). Angew. Chem..

[90] Lin M, Meckes B, Chen C, Teplensky MH, Mirkin CA. 8 (2022). 10.1021/acscentsci.2c00683 PMC9523772 36188351

[91] Mayer Ivy, Karimian Tina, Gordiyenko Klavdiya, Angelin Alessandro, Kumar Ravi, Hirtz Michael, Mikut Ralf, Reischl Markus, Stegmaier Johannes, Zhou Lu, Ma Rui, Ulrich Nienhaus Gerd, Rabe Kersten S., Lanzerstorfer Peter, Domínguez Carmen M., Niemeyer Christof M. (2024). ACS.

[92] Zindel D., Butcher A.J., Al-Sabah S., Lanzerstorfer P., Weghuber J., Tobin A.B., Bünemann M. (2015). Mol. Pharmacol..

[93] Angelin Alessandro, Weigel Simone, Garrecht Ruben, Rebecca Meyer Dr, Bauer Jens, Kapoor Kumar Ravi, Hirtz Dr Michael, Dr Prof, Christof M. (2015). Niemeyer.

[94] Simon Hansen, Jakob C. Stüber, Patrick Ernst, Alexander Koch, Daniel Bojar, Alexander Batyuk & Andreas Plückthun. Sci Rep 7 (2017) 16292. 10.1038/s41598-017-15711-z PMC5701241 29176615

[95] Gandor Silke, Reisewitz Stephanie, Venkatachalapathy Muthukumaran, Arrabito Giuseppe, Reibner Martina, Schröder Hendrik, Ruf Katharina, Niemeyer Christof M., Bastiaens Philippe I.H. (2013). Leif Dehmelt.

[96] Florencia Sánchez M., Faria Sevi, Frühschulz Stefan, Werkmann Lars, Winter Christian, Karimian Tina, Lanzerstorfer Peter, Plochberger Birgit (2024).

[97] Gatterdam Karl, Joest Eike F., Volker Gatterdam Dr (2018). Prof. Dr. Robert Tampé.

[98] Florencia Sánchez M., Dietz Marina S., Müller Ulrike, Weghuber Julian, Gatterdam Karl, Wieneke Ralph, Heilemann Mike, Lanzerstorfer Peter (2022). Robert Tampé.

[99] Wagner R., Stübiger G., Veigel D., Wuczkowski M., Lanzerstorfer P., Weghuber J., Karteris E. (2017). Oncotarget.

[100] Baschieri Francesco, Dayot Stéphane, Elkhatib Nadia, Ly Nathalie, Capmany Anahi, Schauer Kristine, Betz Timo, Matic Vignjevic Danijela, Poincloux Renaud (2018).

[101] Delamarche E., Pereiro I., Kashyap A., Kaigala G.V. (2021). Langmuir.

[102] Velasco V, Shariati SA, Esfandyarpour R. 6 (2020). 10.1038/s41378-020-00185-3 PMC8433138 34567686

[103] Liang Q, Chen Z, Chen X, Huang Q, Sun T. 14 (2023).

[104] Humpel C. Front. Cell. Neurosci. 19 (2025) 1540150. 10.3389/fncel.2025.1540150 PMC11808140 39935610

[105] Steiner K. Humpel C. J Neurosci Methods. 399 (2023) 109979. 10.1016/j.jneumeth.2023.109979 37783349

[106] T.K. D. Chitsaz 12 (2024). 10.1016/j.mex.2024.102665 PMC10957495 38524307

[107] G. Walter, K. Büssow, D. Cahill, A. Lueking and H. Lehrach 3 (2000) 298–302. 10.1016/s1369-5274(00)00093-x 10851162

[108] Shave Steven, Nhan T. (2022). Pham and Manfred Auer.

